# The effects of statins on hyperandrogenism in women with polycystic ovary syndrome: a systematic review and meta-analysis of randomized controlled trials

**DOI:** 10.1186/s12958-021-00863-5

**Published:** 2021-12-20

**Authors:** Jianguo Chen, Chaoran Huang, Tongtong Zhang, Wuqing Gong, Xiaofeng Deng, Hua Liu, Jinbo Liu, Yuanbiao Guo

**Affiliations:** 1 Department of Medical Laboratory, The Affiliated Hospital of Southwest Medical University, Luzhou, Sichuan 646000 China; 2 Department of Clinical Laboratory, Qingbaijiang District People’s Hospital of Chengdu, Chengdu, Sichuan 610300 China; 3 Medical Research Center, The Affiliated Hospital of Southwest Jiaotong University, The Third People’s Hospital of Chengdu, Chengdu, Sichuan 610031 China; 4 Department of Gynecology and Obstetrics, Qingbaijiang District People’s Hospital of Chengdu, Chengdu, Sichuan 610300 China; 5 Neurology Department, The Affiliated Hospital of Southwest Jiaotong University, The Third People’s Hospital of Chengdu, Chengdu, Sichuan 610031 China

**Keywords:** Hyperandrogenism, Hydroxymethylglutaryl-CoA Reductase inhibitors, Polycystic ovary syndrome, Randomized controlled trials, Meta-analysis

## Abstract

Several clinical studies showed that statins were potential to treat polycystic ovary syndrome (PCOS). Through comprehensive search PubMed, EMBASE, the Web of Science, BIOSIS, the ClinialTrails.gov, and the Cochrane Library database up to 14 Feb 2020, we identified the randomized controlled trials about the treatment of statins on hyperandrogenism in PCOS women, and performed a systematic review and meta-analysis. The quality of the included studies was assessed by the Cochrane risk of bias tool and the Jadda score. Subgroup analysis and sensitivity analysis were conducted to analyze the pooled results. Nine trials included 682 PCOS patients were identified. Statins showed a significant potential to reduce testosterone (SMD = -0.47; 95% CI, − 0.76−− 0.18; *P* = 0.002) and dehydroepiandrosterone (SMD = -0.51; 95% CI, − 0.97−− 0.05; *P* = 0.03) levels, compared to the control treatments. The cutaneous symptoms hirsutism (SMD = -0.61; 95% CI, − 1.13−− 0.10; *P* = 0.02) and acne (SMD = -0.92; 95% CI, − 1.49−− 0.34; *P* = 0.002) were significantly improved by statins in PCOS women. Subgroup analysis showed that the two types of statins, and the different control treatments as well, presented no significantly different effect on testosterone and dehydroepiandrosterone. Sensitivity analysis confirmed the stability of the findings from the meta-analysis. In conclusion, statin treatment could significantly reduce androgen levels and improve cutaneous manifestations of hyperandrogenism of PCOS.

## Introduction

Polycystic ovary syndrome (PCOS) is characterized by hyperandrogenism, irregular menses, hirsutism, anovulation, dyslipidemia, hypertension, insulin resistance, and polycystic ovaries when other etiologies are excluded [1–3]. There are 10–15% of reproductive-aged women affected with PCOS [4]. Up to 60–80% of women with PCOS appear hyperandrogenism [1]. Hyperandrogenism is a medical condition characterized by excessive levels of androgens in the periphery or systemically. Hyperandrogenism also corresponds to an important criterion for the diagnosis of PCOS. PCOS symptoms of hirsutism, seborrhea, acne, androgenetic alopecia, and virilization are caused by hyperandrogenism [5–7]. The cutaneous symptoms of hirsutism, acne cause great psychological distress for patients [8, 9]. Pharmacologic treatment is usually used to attenuate PCOS symptoms and prevent long-term adverse effects [10, 11].

Recently, statins emerge a potential to improve the metabolic complications and reproductive endocrine function of PCOS [12–14]. Statins are 3-Hydroxy-3-methylglutaryl-coenzyme A (HMG-CoA) reductase inhibitors and are the first-line drugs to treat hyperlipidemia and dyslipidemia [15]. As steroid hormones derived from cholesterol, statins are considered to inhibit the product of androgens in PCOS patients [16–18]. However, the open clinical randomized controlled trials (RCTs) displayed inconsistent outcomes about the effect of statins on androgens. Some studies showed statins decreased the blood androgens, including testosterone [19–23], dehydroepiandrosterone (DHEA) [19, 24, 25], and androstenedione [25] in PCOS women with hyperandrogenism. Other individual studies however yielded conflicting results with increasing blood androgens or not reaching the statistic difference [21, 23–27].

Ten years ago, a meta-analysis, based on 3 original trials, suggested that statins could reduce testosterone levels in PCOS, but the testosterone levels were not assigned as the primary outcome, and the bias of the trials and stability of the results were not assessed [28]. Another meta-analysis [29] reported that statins could reduce the DHEA levels in PCOS, but it included a nonrandom study [30] in the pooled studies, which limited the reliability of the conclusion. Additionally, those meta-analyses did not pay attention to assessing the effect of statins on the clinical manifestations associated with hyperandrogenism. On the basis of the more RCT studies recently published, we undertook a meta-analysis on the clinical effect of statins on hyperandrogenism, especially the cutaneous symptoms, to obtain more precise evidence of the effects of statins on blood androgens and cutaneous symptoms in women with PCOS.

## Materials and methods

We implemented this study according to the Preferred Reporting Items for Systematic Reviews and Meta-Analyses (PRISMA) guidelines [31].

### Literature search

Two authors (Chen, Deng) independently searched databases including PubMed, EMBASE, the Web of Science, the ClinialTrails.gov, the Cochrane Library, BIOSIS from inception to 14 Feb 2021 to identify relevant published RCTs. The following terms were used for comprehensive literature search: hydroxymethylglutaryl-CoA reductase inhibitors, Polycystic Ovary Syndrome (as MeSH terms) combined with statins*, fluvastatin, pravastatin, lovastatin, simvastatin, atorvastatin, rosuvastatin, lipid-lowering drugs, Sclerocystic Ovaries, ovary polycystic disease (as text words). We also performed a manual retrieval of the reference cited in the reviews and meta-analyses. The initial search results were checked for any duplicate publications by titles and abstracts, and then we screened articles according to the inclusion criteria to identify the most relevant studies. We excluded the case reports, editorials, letters to the editor, retrospective studies, and review articles. After excluding the nonrelevant articles by screening titles and abstracts, a further detailed review of the full text was conducted to carry out the final qualitative and quantitative analysis. Ethical approval is not required because no patient was recruited or personal information was collected.

### Study eligibility and exclusion criteria

Eligible studies were considered to meet the following criteria: studies assessed the effects of statins on androgen levels or manifestations of hyperandrogenism in women with PCOS; randomized clinical trials; studies that used statins continued for at least 1 week; studies that reported adequate information to extract data we interested; the full text of the article was available to acquire. The exclusion criteria included interventional studies without the appropriate control treatment, studies lacking adequate baseline or post-intervention data, and studies that were not written in English. When duplicate data of the same study were found, we choose the publication which had the maximum population or duration of treatment.

### Data extraction

Two reviewers (Chen and Gong) extracted the details for each study independently; if disagreement occurred, two authors discussed and arrived at a consensus with a third author (Guo), data extracted included: name of the first authors; publication year; the country where the study was performed; sample capacity; study design; patient characteristics; rates and reasons of drop out from the study, the type of statin, dose and duration of statin use; mean and standard deviation (SD) of the change in androgen levels and manifestations of hyperandrogenism during the trial.

### Quality evaluation

Two independent authors (Zhang and Liu) evaluated the quality of the eligible studies using the Cochrane risk of bias tool for RCTs [32] and the Jadad score [33]. The assessment of quality by the Cochrane risk of bias tool includes random sequence generation, allocation concealment, blinding of participants and personnel, blinding of outcome data, incomplete outcome data, and selective reporting. The overall Jadad score ranges from 0 to 5 points based on random sequence, blind method, lost to follow-up, and withdrawal of included studies. The higher scores represented the higher the quality of the study.

### Statistical analysis

In this meta-analysis, we assessed several outcomes. The change of testosterone level was the primary outcome; other outcomes included changes of DHEA level, hirsutism score, acne score. All outcomes extracted from the literature were continuous data. The outcomes were presented as mean values and SD. Considering the different units of the included studies, we choose standardized mean difference (SMD) and 95% confidence interval (CI) to assess the degree of the effects, the effect with *P* < 0.05 was considered to be statistically significant. When studies did not directly report the SD or variance, the variance was calculated using the 95% CI. The Q-test and the I^2^ index were used to assess study heterogeneity. Q-test with *P* < 0.1 or I^2^ values≤50% represents statistical homogeneity [34], we chose the fixed-effect model; otherwise, we chose the randomized-effect model [35]. To explore the origin of heterogeneity, We performed subgroup analysis based on the statin type and therapy of the control group. However, the pooled analyses on subgroups were carried out only when there were at least 2 studies in each subgroup; sensitivity analysis was performed by sequentially removing one study at a time. Publication bias was assessed by funnel plot analysis. All the analytic process was performed by Revman 5.4 software (Nordic Cochrane Centre, Cochrane Collaboration).

## Results

### Literature search

**Fig. 1:**
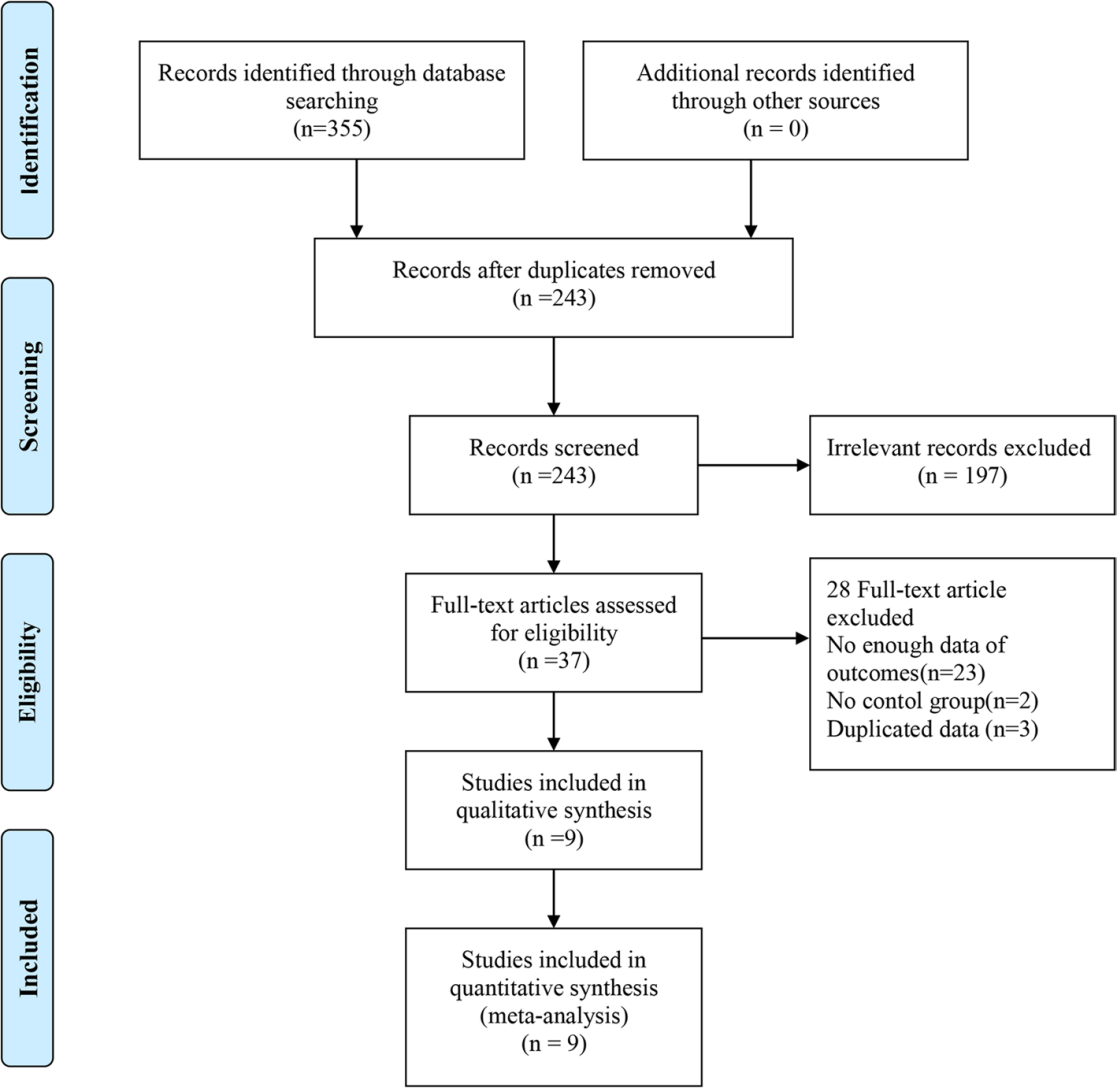
Flowchart of the selection of studies

We identified 243 RCTs through initial database searches. According to our purpose, we excluded 197 irrelevant studies by screening the titles and abstracts. Among the remaining 37 trials, 28 publications were further excluded due to the absence of outcome details (*n* = 23), no appropriate control group (*n* = 2), or duplicated data (*n* = 3). Finally, 9 studies were screened out for the meta-analysis. A flowchart of the study selection is shown in Fig. 1**.**

### Study characteristics and quality evaluation

**Fig. 2:**
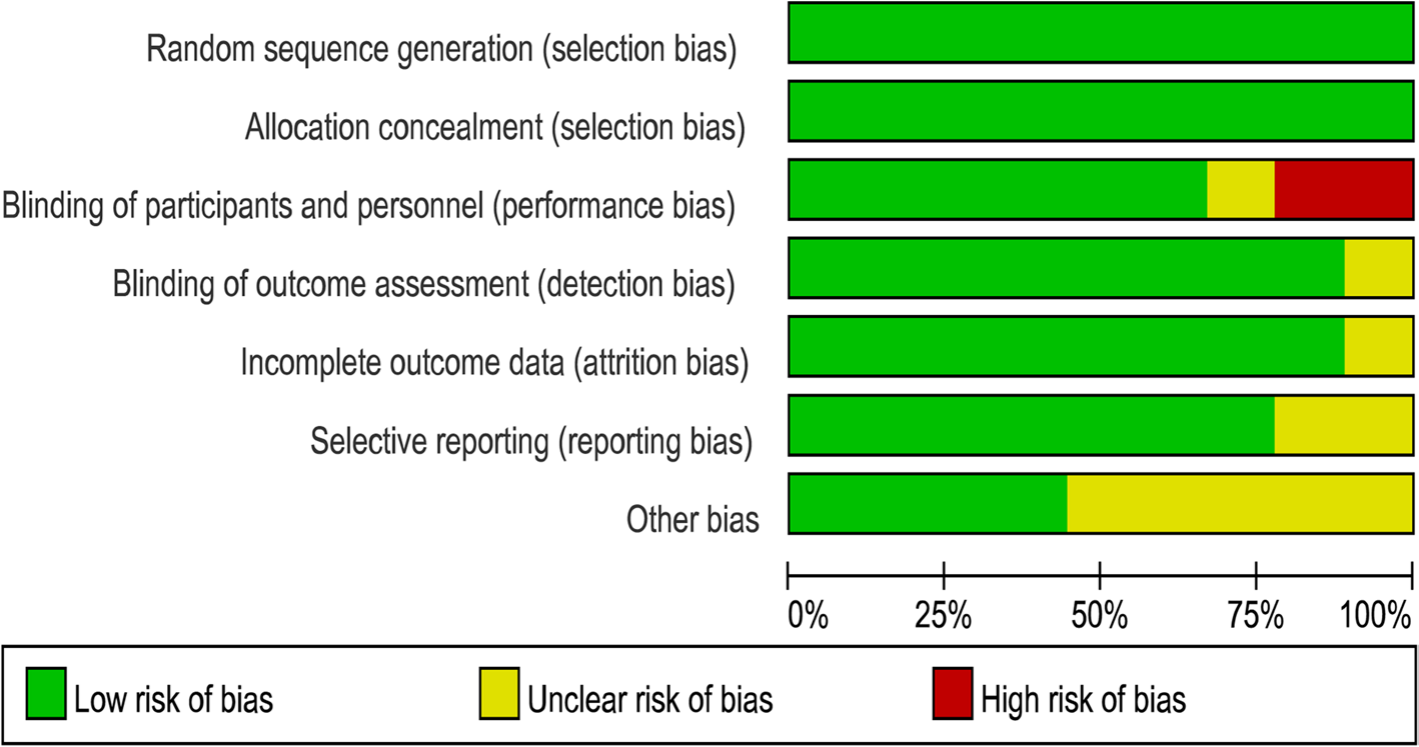
Risk of bias

**Fig. 3:**
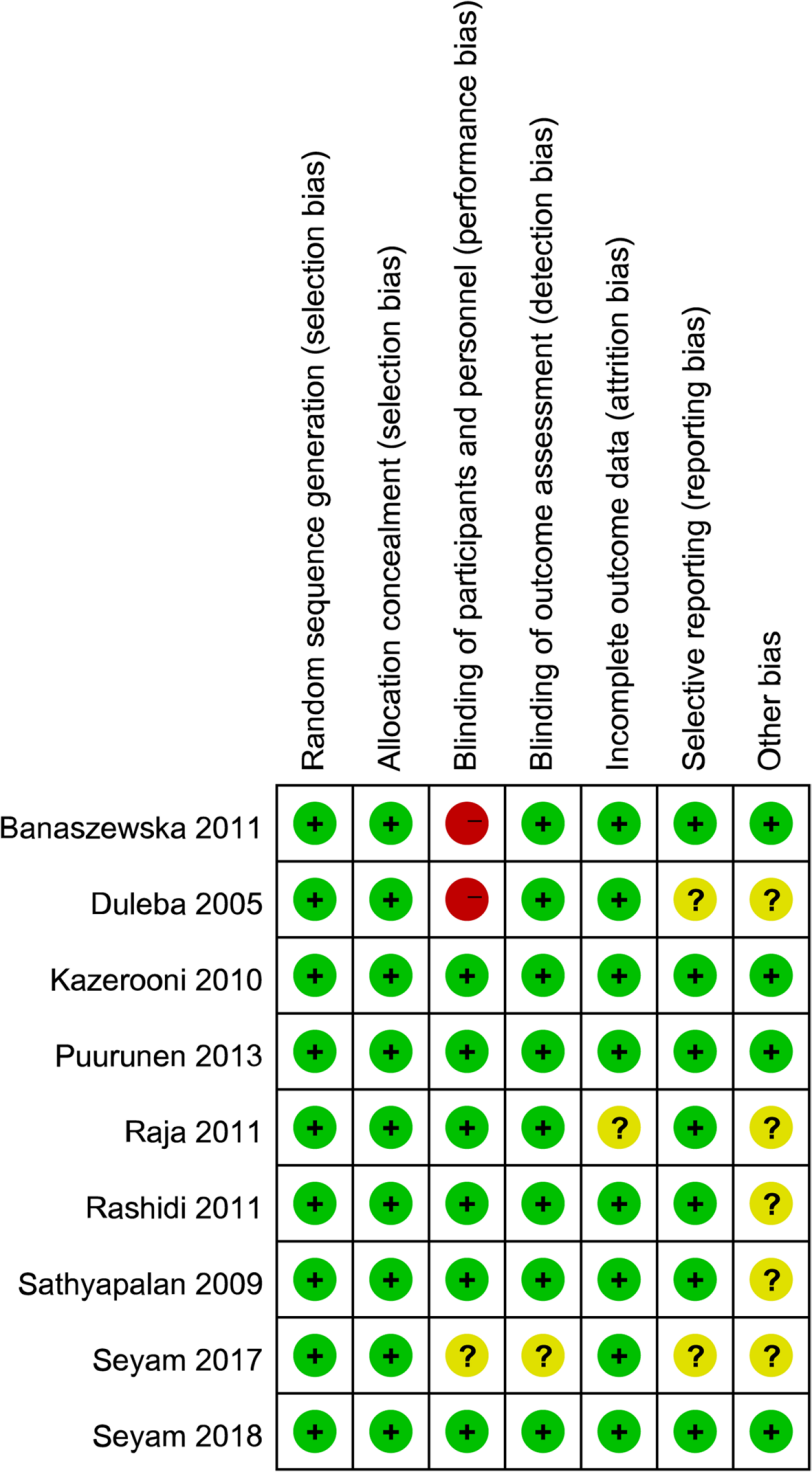
Summary of risk of bias

In the 9 RCTs, 347 out of 682 PCOS patients were involved in the statin treatment group and the other 335 patients in the control treatment group. Two kinds of statins were used in the 9 RCTs, simvastatin for 6 studies [19–21, 23, 24, 27] and atorvastatin for three studies [22, 25, 26, 36]. About the controls, placebo was used for five studies, metformin for 3 studies, oral contraceptive pills (OCP) for 1 study. The specific characteristics of all 9 studies are summarized in Table 1. The median points of the Jadad score were 4, and the findings of the quality of each trial were evaluated by the Cochrane risk of bias tool is shown in Figs. 2 and 3.

**Table 1:** Characteristics of the studies included in the meta-analysis

Publication author, year	Country	Jadad score	Treatment arm A	Treatment arm B	Time of therapy	Sample size	Outcomes	Assessment method/Unit of primary outcome
Seyam 2018 [19]	Egypt	5	Simvastatin(20 mg/d) +Metformin(1.5 g/d)	Metformin(1.5 g/d)	12 months	70/65	T,DHEA, Hirsutism	ELISA assay/ ng/ml
Seyam 2017 [24]	Egypt	4	Simvastatin(20 mg/d)	Placebo	6 months	100/100	T,DHEA, Hirsutism,Acne	chemiluminescence assays/ ng/ml
Puurunen 2013 [26]	Finland	5	Atorvastatin 20 mg/d	Placebo	6 months	15/13	T,DHEA, And	Liquid mass spectrometry/ nmol/l
Sathyapalan 2009 [22]	United Kingdom	5	Atorvastatin 20 mg/d	Placebo	3 months	19/18	T	Immunoassay/ nmol/l
Banaszewska 2011 [27]	Poland	3	Simvastatin(20 mg/d) +Metformin(1.7 g/d)	Metformin(1.7 g/d)	6 months	36/33	T,DHEA, Hirsutism,Acne	enzymatic colorimetric assays/ ng/ml
Raja 2011 [25]	American	4	Atorvastatin(40 mg/d)	placebo	1.5 months	9/11	T,DHEA,And Hirsutism,Acne	not report/ ng/dl
Rashidi 2011 [20]	Iran	5	Simvastatin(20 mg/d)	placebo	2 months	32/29	T,DHEAS	chemiluminescence assays/ pg/ml
Kazerooni 2010 [21]	Iran	5	Simvastatin(20 mg/d) +Metformin(1.5 g/d)	Metformin(1.5 g/d)	3 months	42/42	T,DHEA,Hirsutism	Radioimmunoassay/ ng/ml
Duleba 2005 [23]	Poland	2	simvastatin, 20 mg +OCP	OCP	3 month	24/24	T,DHEA	chemiluminescence assays/ ng/dl

*T* testosterone, *DHEA* dehydroepiandrosterone, *And* androstenedione, *OCP* oral contraceptive pills; containing 20 μg ethinyl E2 and 150 μg desogestrel

### Effects of statins therapy on androgens levels

**Fig. 4:**
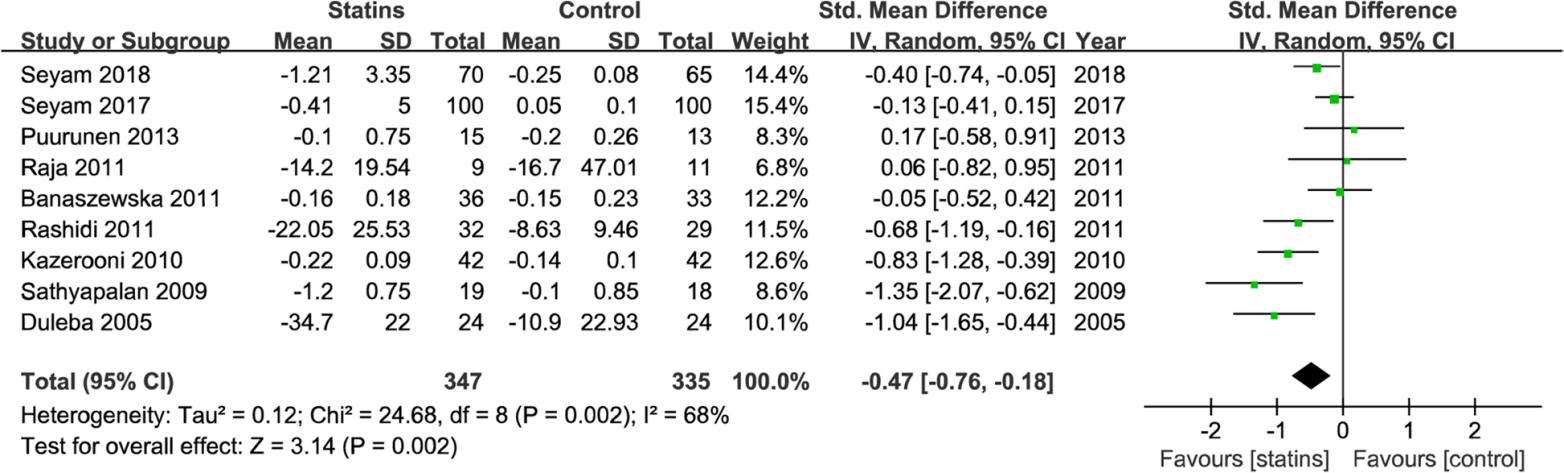
Forest plot of the effect of statins on the levels of testosterone

**Fig. 5:**
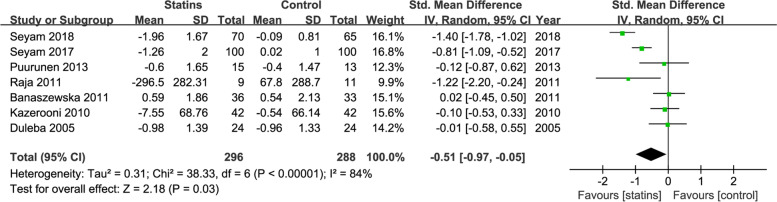
Forest plot of the effect of statins on the levels of dehydroepiandrosterone (DHEA)

**Fig. 6:**
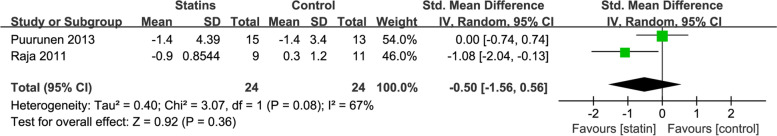
Forest plot of the effect of statins on the levels of androstenedione

We choose random-effects model because substantial heterogeneities were observed. Effects of statins on Androgens include testosterone, DHEA, androstenedione were analyzed in the pooled studies. Meta-analysis showed that compared with control treatment, statins reduced blood testosterone levels in nine studies (SMD = -0.47; 95% CI, − 0.76−− 0.18; *P* = 0.002, I^2^ = 68%) (Fig. 4). DHEA levels were reduced by statins in seven studies (SMD = -0.51; 95% CI, − 0.97−− 0.05; *P* = 0.03; I^2^ = 84%)(Fig. 5), but two studies reported androstenedione change were not reach the statistic difference (SMD = -0.50; 95% CI, − 1.56 − 0.56; *P* = 0.36; I^2^ = 67%) (Fig. 6) .

### Effects of statin therapy on cutaneous manifestations

**Fig. 7:**
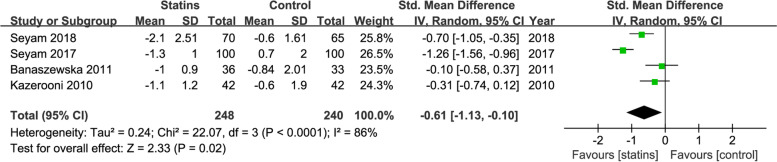
Forest plot of the effect of statins on the hirsutism

**Fig. 8:**
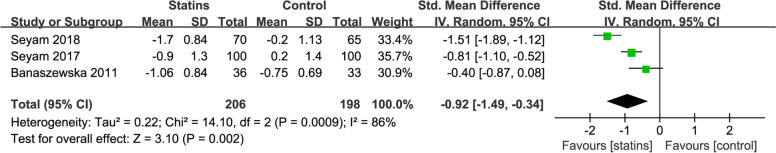
Forest plot of the effect of statins on acne

Four studies provided data on the change in hirsutism score, and 3 provided data on ance score. Results show that compared to control treatment, statin treatment could relieve the manifestations of hirsutism (SMD = -0.61; 95% CI, − 1.13−− 0.10; *P* = 0.02; I^2^ = 86%)(Fig. 7) and acne (SMD = -0.92; 95% CI, − 1.49−− 0.34; *P* = 0.002, I^2^ = 86%) (Fig. 8).

### Subgroup analysis

There is no significant difference between the effects on testosterone of the two control treatments placebo (SMD = -0.39; 95%CI, − 0.86 − 0.09; *P* = 0.11; I^2^ = 71%) or metformin (SMD = -0.43; 95%CI, − 0.84−− 0.02; *P* = 0.04; I^2^ = 65%) implied in the 9 studies. The two statins simvastatin (SMD = -0.48; 95%CI, − 0.77−− 0.18; P = 0.002; I^2^ = 66%) and atorvastatin (SMD = -0.39; 95% CI, − 1.38 − 0.61; *P* = 0.45; I^2^ = 80%) also revealed no significant difference in reducing testosterone and DHEA (Table 2). We did not conduct subgroup analysis in effects of statins on androstenedione and cutaneous manifestations, because less than 2 studies were in each subgroup.

**Table 2:** Subgroup analysis in testosterone and DHEA

Subgrouped by	No. of trials	WMD (95% CI)	*P* Value	P for heterogeneity	I^2^ (%)	P for between Subgroup heterogeneity
**Testosterone**						
total	9	-0.47[−0.76,-0.18]	0.002	0.002	68	
Statin type						0.86
Simvastatin	6	-0.48 [−0.77, − 0.18]	0.002	0.002	66	
Atorvastatin	3	−0.39 [−1.38, 0.61]	0.45	0.007	80	
Contol type						0.90
Placebo	5	-0.39 [−0.86, 0.09]	0.11	0.008	71	
Metformin	3	−0.43 [− 0.84, − 0.02]	0.04	0.06	65	
**DHEA**						
total	7	−0.51 [− 0.97, − 0.05]	0.03	< 0.001	84	
Statin type						0.79
Simvastatin	5	−0.46 [−1.02, 0.09]	0.10	< 0.001	89	
Atorvastatin	2	−0.62 [−1.70, 0.45]	0.25	< 0.001	67	
Contol type						0.70
Placebo	3	−0.49 [−1.44, 0.46]	0.31	< 0.001	93	
Metformin	3	−0.70 [−1.20, − 0.21]	0.005	0.15	47	

### Sensitivity analysis and publication bias

**Fig. 9:**
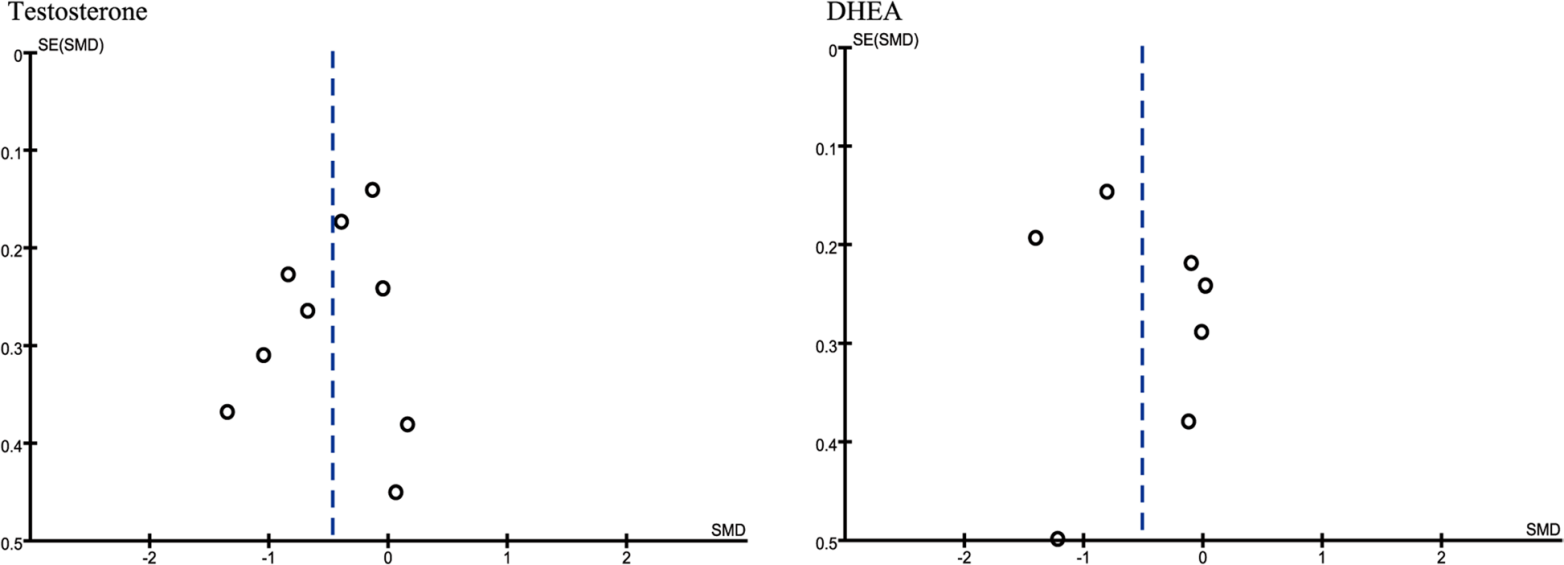
Funnel plot of the studies included in the meta-analysis

Sensitivity analysis was performed to evaluate the stability of the meta-analysis. When any single study was removed, the overall statistical significance did not change in testosterone with SMD range from − 0.53(95CI, − 0.85 − 0.21) to − 0.39 (95CI, − 0.66 − 0.12), and DHEA with SMD range from − 0.61 (95CI, − 1.10−− 0.17) to − 0.33 (95CI, − 0.73 − 0.06), which indicated that the results of this meta-analysis were relatively stable (Table 3). The study publication bias was assessed by funnel plot, which showed no significant bias in testosterone and DHEA (Fig. 9). We did not perform sensitivity analysis and publication bias assessment in other outcomes because less than 5 studies were included.

**Table 3:** Sensitivity analysis of Testosterone and DHEA

Excluded study	Testosterone		DHEA	
	SMD	95CI	SMD	95CI
None	−0.47	[−0.76,-0.18]	− 0.51	[− 0.97,-0.05]
Seyam 2018	− 0.48	[− 0.84,-0.13]	−0.33	[− 0.73,0.06]
Seyam 2017	−0.53	[− 0.85, 0.21]	−0.46	[−1.04, 0.13]
Puurunen 2013	−0.53	[−0.83, 0.22]	− 0.57	[−1.07,-0.06]
Banaszewska 2011	−0.53	[− 0.85, 0.21]	−0.61	[−1.10,-0.12]
Raja 2011	−0.51	[−0.81, 0.20]	− 0.43	[− 0.92,0.06]
Rashidi 2011	− 0.44	[− 0.76, 0.12]	–	–
Kazerooni 2010	− 0.41	[− 0.72, 0.11]	−0.59	[− 1.09,-0.09]
Sathyapalan 2009	−0.39	[− 0.66, 0.12]	–	–
Duleba 2005	−0.40	[− 0.70, 0.11]	− 0.60	[− 1.05,-0.16]

## Discussion

Statins appear to be safe, especially for long-term use. They are widely used to improve hyperlipidemia and protect from long-term cardiovascular morbidity [37]. This article provided evidence that using statins may offer additional benefits for women with PCOS by improving hyperandrogenism.

Years ago, two reviews employed few RCTs and implied a positive effect of statin treatment reducing testosterone and DHEA levels of PCOS women [28, 29]. Now, more RCTs included in our meta-analysis made this conclusion solid. Compared with the previous reviews, our findings provided the most up-to-date evidence on this topic and expanded the sample size, which enhanced statistical power and provided more precise and reliable results. Through broad search strategy and system review, our study had amended several limitations in previous reviews. This systematic review and meta-analysis of randomized controlled trials combined the outcomes of 682 women with PCOS from 9 individual studies, indicating that statins can reduce the levels of testosterone and DHEA. However, there was no statistical change about androstenedione in statin treatment, possibly attributed to the small sample size with only 48 patients pooled from two trials.

Cutaneous symptoms hirsutism of PCOS are complications due to the combined effect of excessive androgen and the sensitive response of the hair follicle. Up to 75% of PCOS women with hirsutism have measurable hyperandrogenemia [38], however, suppression of androgen production by oral contraceptives, the first-line treatment, is not so effective. Moreover, the severity of hirsutism is not well-correlated with the inordinate level of androgen excess, though the cutaneous symptoms of PCOS are theoretically a result of hyperandrogenism. It indicates the intricacy of those cutaneous comorbidities. Cumulating evidence shows that a single agent is not satisfactory, and combination therapy is recently recommended [39, 40]. Statins are effective for reducing androgen levels in PCOS women, but their use to control skin comorbidities is still uncertain. A previous meta-analysis displayed that there was insufficient evidence supporting the therapeutic efficacy of statins on hirsutism in premenopausal women [41]. With the addition of the recent RCTs, our meta-analysis demonstrated that statins could relieve cutaneous symptoms of hirsutism and acne. Simvastatin presented more active treatment on both hirsutism and acne than metformin, probably on account of DHEA sulphate (DHEAS) that inhibited by simvastatin while elevated by metformin [27]. A meta-analysis included 55 study groups with a total of 6593 PCOS patients documents that DHEAS and androstenedione are positively associated with Ferriman-Gallwey (FG) score, which is well accepted as a gold standard for hirsutism diagnosis to determine the density of terminal hairs at nine different body sites [38]. Whereas the other biochemical hyperandrogenism parameters including total testosterone, free testosterone, free androgen index, sex hormone binding globulin (SHBG) are negatively associated with FG scores, although the effects of simvastatin or metformin on SHBG is akin to DHEAS [27]. In fact, serum concentrations of DHEA and DHEAS, rather than testosterone, are significantly higher in axillary hair-positive than in -negative women over 50 years old [42]. Furthermore, oral administration of DHEA to young females with central adrenal insufficiency or adult hypopituitary women exhibits significant progress in pubic and/or axillary hair growth [43]. However, this effect of DHEA needs more experimental evidence to understand the mechanism, because DHEA inhibits hair follicle growth and blocks the transition from telogen to anagen in gonadectomized male and female mice [44].

Acne affects 15–25% of PCOS women [10]. Acne score declines over 60% after oral or topical statin treatment for patients with or without PCOS [27]. Reviewed the published knowledge, we also find lessening DHEA is probably a reason why statins are more capable of acne than metformin. DHEAS in scalp hair and blood are higher in acne women than in the normal controls, but serum testosterone levels are comparable in the two populations [45, 46]. Moreover, serum DHEAS is correlated to the severity of acne, and its increase implies an aggravating effect on the risk of acne [45, 47]. DHEAS is considered to be a key role in the pathogenesis of adult-onset acne [48].

Although the target gene of statins is 3-Hydroxy-3-methyl-glutaryl-coenzyme A reductase (HMG-CoA reductase), the rate-limiting enzyme of cholesterol production, and is responsible for cholesterol metabolism of skin sebaceous glands and hair follicles [49]. However, the mechanisms that undertake the therapeutic effect of statins on hirsutism are enigmatic. In contrast to this, several reports display the therapeutic potential of statins for hair loss [50]. Simvastatin and atorvastatin are commonly used drugs with different effectiveness in lowering blood lipid levels and fat solubility, an animal study reported that statins vary greatly with regard to their effects on steroidogenesis of rat ovarian theca-interstitial cells. Compare to atorvastatin, simvastatin may exert greater inhibitory effects on the number of viable cells and production of androgens [51], a RCT also indicated simvastatin was more effective in reducing the serum testosterone level (by 27% vs. 16%) [52]. However, on subgroup analysis, the two kinds of statins showed similar effects on hyperandrogenism in women with PCOS, the effects of different statins on hyperandrogenism need more studies to certain, In addition, we performed sensitivity analyses, which confirmed the robustness of the main results.

However, there are some limitations in this meta-analysis. First, the heterogeneity among the studies probably limited the credibility of the study, but we did not find the origin of heterogeneity partially because of few RCTs included. Second, the levels of androgens were measured by different methods in the various study, which might result in incomparability and heterogeneity between the data from different RCTs. Third, the score of cutaneous manifestation of hirsutism and acne was subjective based on various standards. Finally, this study was constrained to studies published in English only. So publication bias cannot be excluded.

## Conclusion

This systematic review and meta-analysis of 9 RCTs indicated that statins could reduce the levels of androgens and improve the cutaneous manifestations of hyperandrogenism in women with PCOS, providing evidence for supporting the statins as a potential treatment for women with PCOS. It appears crucial to confirm the additional beneficial effects of statins on hyperandrogenism when aiming to improve health conditions for women with PCOS. To achieve this, more high validity trials, with more comprehensive outcomes of hyperandrogenism and more focus on the subgroups are required to explore the origin of heterogeneity and determine the effects of statins on hyperandrogenism.

## Data Availability

The datasets used and/or analysed during the current study are available from the corresponding author on reasonable request.
