# Correction to: The effects of statins on hyperandrogenism in women with polycystic ovary syndrome: a systematic review and meta-analysis of randomized controlled trials

**DOI:** 10.1186/s12958-022-00941-2

**Published:** 2022-04-15

**Authors:** Jianguo Chen, Chaoran Huang, Tongtong Zhang, Wuqing Gong, Xiaofeng Deng, Hua Liu, Jinbo Liu, Yuanbiao Guo

**Affiliations:** 1 Department of Medical Laboratory, The Affiliated Hospital of Southwest Medical University, Luzhou, 646000 Sichuan China; 2 Department of Clinical Laboratory, Qingbaijiang District People’s Hospital of Chengdu, Chengdu, 610300 Sichuan China; 3 Medical Research Center, The Affiliated Hospital of Southwest Jiaotong University, The Third People’s Hospital of Chengdu, Chengdu, 610031 Sichuan China; 4 Department of Gynecology and Obstetrics, Qingbaijiang District People’s Hospital of Chengdu, Chengdu, 610300 Sichuan China; 5 Neurology Department, The Affiliated Hospital of Southwest Jiaotong University, The Third People’s Hospital of Chengdu, Chengdu, 610031 Sichuan China

**Correction to: Reprod Biol Endocrinol 19, 189 (2021)**

**https://doi.org/10.1186/s12958-021-00863-5**

Following publication of the original article [1], the authors reported an error to affiliation of author Yuanbiao Guo wherein affiliation 1 was inadvertently removed during correction stage. Author Yuanbiao Guo should be affiliated to:

Department of Medical Laboratory, The Affiliated Hospital of Southwest Medical University, Luzhou, Sichuan 646000, China.

Medical Research Center, The Affiliated Hospital of Southwest Jiaotong University, The Third People’s Hospital of Chengdu, Chengdu, Sichuan 610031, China.

The original article [1] has been updated.
